# Structural biology in discovery and development of heterobifunctional degraders

**DOI:** 10.1063/4.0001100

**Published:** 2025-10-27

**Authors:** Kunhua Li

**Affiliations:** Kymera Therapeutics, Watertown, MA, USA

## Abstract

Structural biology has been a key component in structure-based drug design and is crucial in lead discovery and optimization. While numerous studies highlight how binary protein-compound structures have influenced the creation of innovative chemical matters in protein degradation, enabling ternary complex structures to further expand structural biology’s impact to accelerate the rational design and mechanistic understanding of the degrader molecules. At Kymera, our structural biology group has integrated various structural biology techniques, including X-ray crystallography, MicroED, and Cryo-EM, into our discovery pipelines to pioneer targeted protein degradation and to invent new medicines. Here, we present two case studies in discovering and developing the heterobifunctional (HBF) degraders for IRAK4 and CDK2. Guided by X-ray crystallography, we have designed a novel protein of interest (POI) and cereblon (CRBN) binders with improved selectivity. For HBF degraders, we have solved the high-resolution Cryo-EM structures of the ternary complexes, confirmed that our degraders selectively recruited the POI to the CRBN, which lead to the ubiquitination of the POI or POI complex for subsequential degradation. These case studies demonstrated the impact of structural biology in developing HBF degraders with robust in vivo efficacy.

